# Aging increases the oxidation of dichlorohydrofluorescein in single isolated skeletal muscle fibers at rest, but not during contractions

**DOI:** 10.1152/ajpregu.00530.2012

**Published:** 2013-05-22

**Authors:** Jesus Palomero, Aphrodite Vasilaki, Deborah Pye, Anne McArdle, Malcolm J. Jackson

**Affiliations:** Institute of Ageing and Chronic Disease, University of Liverpool, Liverpool, United Kingdom

**Keywords:** muscle, reactive oxygen, single fiber

## Abstract

An increase in the activity of reactive oxygen species (ROS) has been implicated in the mechanisms of loss of skeletal muscle that occurs during aging, but few studies have attempted to directly assess activities in intact muscle fibers. The current project used the nonspecific fluorescent probe for ROS and reactive nitrogen species, 5-(and-6)-chloromethyl-2′,7′-dichlorodihydrofluorescein (CM-DCFH), in single, isolated, mature skeletal muscle fibers from adult and old mice in addition to biochemical measurements of key regulatory proteins for ROS in muscles of these animals. Data confirmed the changes in key regulatory processes for ROS (increased glutathione peroxidase 1 and catalase activities and reduced total glutathione content) previously reported in muscle from old mice and showed increased CM-DCFH oxidation in muscle fibers from old mice at rest and indicate that these changes are likely due to an increase in generation of oxidants rather than a lack of scavenging capacity. The increased CM-DCFH oxidation persisted even when cellular defenses against oxidants were increased by loading fibers from young and old mice with glutathione. During contractile activity, and in contrast to the increase observed in fibers from young mice, there was no further increase in CM-DCFH oxidation in muscle fibers from old mice. These data also suggest that the defect in short-term adaptations to contractions that occurs in old mice may be related to a diminished, or absent, increase in the muscle generation of ROS and/or reactive nitrogen species that normally accompanies contractile activity in young mice.

in older people, declining muscle mass and function lead to instability, increased risk for falls, and residential care (60). By age 70, the cross-sectional area of skeletal muscle is reduced by 25–30%, and muscle strength is reduced by 30–40% (43). The reduction in muscle mass and function with age in humans and rodents is primarily due to a decrease in the number of muscle fibers, and atrophy and weakening of those remaining (4, 28, 29). The loss of muscle fibers and other changes in muscle during aging show considerable similarities between humans and rodents (34). Advancing age is associated with other functional changes in the remaining muscle fibers, including a slowing of the muscle phenotype (9) and an attenuation of important responses to contractions that include acute stress responses (56), mitochondrial biogenesis (30), and anabolic responses (8). Correction of specific attenuated responses to contractions has been found to preserve muscle force generation in old mice (5, 27, 31).

An age-related increase in the activities of free radicals and reactive oxygen species (ROS) has been implicated in the fundamental processes underlying aging, and many early studies indicated that ROS were inevitably deleterious to cells, causing oxidative damage to lipids, DNA, and proteins (17), but it is now recognized that in normal physiology, ROS mediate many adaptive processes following physiological stresses. ROS are important physiological signaling molecules with regulatory functions that modulate changes in cell and tissue homeostasis and gene expression (12, 15, 22). Signaling by these reactive molecules is achieved mainly by targeted modifications of specific residues in proteins (25). Skeletal muscle fibers respond to contractile activity by increasing the intracellular generation of superoxide and nitric oxide (NO), with the formation of secondary ROS and reactive nitrogen species (41, 44, 45).

In all species, tissues (including skeletal muscle) of aged organisms contain oxidative damage to lipids, DNA, and proteins compared with that found in young organisms (11, 50, 55); also, the hypothesis that increased oxidative damage plays a key role in age-related tissue dysfunction has been extensively examined. In nonmammalian models, some transgenic or pharmacological interventions designed to reduce the activities of ROS (33, 37–39) extended lifespan, but these effects are not universally observed and are controversial (14). In mammals, few genetic manipulations designed to reduce ROS activities have resulted in increased lifespan [e.g., (51, 59)]. Many studies have reported that ROS generation is increased in mitochondria isolated from skeletal muscle of aged animals [see Van Remmen and Jones (53) for a review] and that this occurs in association with impaired mitochondrial function and oxidative damage to mitochondrial components (23). There are also some studies that indicate manipulation of ROS activities can preserve muscle function during aging (5, 51).

Few studies have attempted to monitor reactive oxygen or nitrogen species in intact skeletal muscle of aged organisms, although muscle from aged rodents contains increased amounts of oxidative damage (e.g., see Refs. 5 and 55). One potential approach is to use fluorescent probes that are sensitive to reactive oxygen and nitrogen species, and Ji and colleagues (2, 3) reported increased dichlorohydrofluorescein (DCFH) oxidation in homogenates of tissues from old compared with young rats, but the data from such studies are potentially influenced by differential effects of the homogenization procedure on old compared with young tissue. We have previously described the use of the chloromethyl derivative of DCFH (CM-DCFH) to obtain a measure of reactive oxygen and nitrogen species in isolated cultured single skeletal muscle fibers during contractile activity or passive stretching (40, 41). This approach has the advantage of specifically studying skeletal muscle fibers in the absence of contaminating cells (such as endothelial or white cells), but DCFH-based probes are acknowledged to react with a variety of reactive oxygen and nitrogen species e.g., hydrogen peroxide (H_2_O_2_), organic peroxides, hydroxyl radical, NO, and peroxynitrite (36).

We hypothesized that in single fibers from muscles of old mice, the oxidation of the nonspecific probe CM-DCFH would be increased compared with the oxidation in fibers from young mice. Furthermore, we additionally hypothesized that this oxidation would be increased by contractile activity in fibers from both young and old mice. This study therefore examined CM-DCFH oxidation in single isolated fibers from old compared with young mice, both at rest and following a period of contractile activity. The data obtained indicate that differences occur between fibers from aged and young mice; a secondary aim was to modify the oxidative status of fibers from both old and young mice ex vivo to attempt to understand factors that might contribute to the changes in CM-DCFH oxidation observed. Fibers from both groups were therefore stressed by treatment with physiologically relevant amounts of H_2_O_2_, or treated with glutathione ethyl ester to increase the glutathione content of fibers, and hence increase their ability to scavenge oxidants, while examining rates of CM-DCFH oxidation.

## MATERIALS AND METHODS

### 

#### Mice.

Experiments were performed in accordance with U.K. Home Office Guidelines under the U.K. Animals (Scientific Procedures) Act 1986, and received ethical approval from the University of Liverpool Animal Welfare Committee. C57Bl6 female mice (young mice, 2- to 4-mo; old mice were 26–28 mo) were used in this study. Animals were maintained in a temperature-controlled environment and fed a standard laboratory chow diet ad libitum and subjected to a 12-h light-dark cycle.

Mice were killed by an overdose of anesthetic (ketamine hydrochloride and medatomidine hydrochloride) by intraperitoneal injection. Both tibilais anterior and gastrocnemius muscles were removed, weighed, and stored at −80°C for further analysis, and the flexor digitorum brevis (FDB) muscles were removed for isolation of intact single fibers.

#### Isolation of single mature skeletal muscle fibers.

Single muscle fibers were isolated from the FDB muscles of mice as previously described (41, 45). Muscles were placed into 0.4% type I collagenase (EC 3.4.24.3; Sigma-Aldrich, Dorset, UK) solution in the culture medium. This was composed of minimum essential medium (MEM; Sigma-Aldrich) supplemented with 10% fetal bovine serum (FBS) (Invitrogen, Paisley, UK) containing 2 mM glutamine, 50 IU penicillin, and 50 μg/ml streptomycin. Both FDB muscles from each mouse were incubated in collagenase solution at 37°C for 2 h, and the mixture was manually shaken every 30 min to improve digestion of connective tissue. Fiber bundles that had not been separated during incubation were gently triturated by a wide-bore plastic pipette to separate fibers. Free single muscle fibers were separated from broken fibers and single cells by centrifuging at low speed (600 *g* for 30 s) four times. After each centrifugation, the supernatant was removed and replaced with fresh culture medium. Washed fibers were plated onto a 35-mm culture plate that had been previously coated with a collagen matrix (Matrigel, BD Biosciences) and incubated for 18–24 h covered with culture medium at 37°C in 5% CO_2_ in a humidified atmosphere to allow adherence of the fibers to Matrigel. Experiments were performed only on fibers that displayed excellent morphology and exhibited clear striations along the sarcolemma.

#### Loading of fibers with fluorescent probes.

5-(And-6)-chloromethyl-2′,7′-dichlorodihydrofluorescein diacetate (CM-DCFH-DA) (Molecular Probes, Invitrogen) was used as a general probe for reactive oxygen and nitrogen species. After 18–24 h incubation to allow fiber attachment to the Matrigel, plates with fibers were washed with Dulbecco's phosphate buffered saline (D-PBS) and loaded with CM-DCFH-DA (17.5 μM) in D-PBS for 30 min at 37^○^C (41). After CM-DCFH-DA loading and 30 min of incubation, fibers were washed twice with D-PBS and Eagle's minimum essential medium without phenol red (to avoid interference with fluorescence imaging) was added to fibers to maintain these cells during the fluorescence microscopy imaging.

5-Chloromethylfluorescein diacetate (CMFDA) (CellTracker Green CMFDA; Molecular Probes, Invitrogen) was used to monitor intracellular glutathione (GSH) (18, 26, 52). For CMFDA loading, fibers were incubated with CMFDA 5 μM in D-PBS for 30 min, then washed twice with D-PBS and maintained in MEM without phenol red for fluorescence microscopy. CMFDA is permeable and crosses the plasma membrane. Once inside the cell, cytoplasmic esterases convert CMFDA into a nonfluorescence impermeable molecule (CMF). This reacts with GSH and fluoresces green when excited with blue light. The fluorescence (CMF fluorescence) is monitored by epifluorescence microscopy.

#### Microscopy and fluorescent imaging.

The imaging system consisted of a Zeiss Axiovert 200M epifluorescence microscope equipped with an 500/20 excitation 535/30 emission filter set for the detection of CM-DCF fluorescence. With the use of a ×20 objective, fluorescence images were captured with a computer-controlled Zeiss MRm charged-coupled device camera (Carl Zeiss) and analyzed with the Axiovision 4.0 image capture and analysis software (Carl Zeiss Vision). All experiments were carried out at 25°C.

#### Contractile activity induced by electrical stimulation.

Contractions in single isolated muscle fibers were induced by electrical field stimulation using established techniques (32, 41, 45). After loading was completed, fibers remained at rest for 15 min and were then exposed to trains of bipolar square wave pulses of 2 ms duration for 0.5 s every 5 s at 50 Hz and 30 V/well. This lasted for 15 min, and then fibers remained at rest for the duration of the experiment. Fibers were observed throughout the contraction period and only those fibers that contracted throughout were used for analyses.

#### Treatment of fibers with H_2_O_2_ or glutathione ethyl ester.

Plates of fibers were treated with 5 μM H_2_O_2_ for 30 min during the period 15–45 min after commencing the measurements of CM-DCF fluorescence. Other plates were pretreated with 1 mM glutathione ethyl ester (GSHEE) for 18 h prior to loading with CM-DCFH as previously described (39).

#### Analysis of muscle contents of total glutathione, oxidized glutathione, glutathione peroxidase activity, catalase activity, and total superoxide dismutase activity.

Assays were undertaken on homogenates of the gastrocnemius muscles from young and old mice. Total GSH and disulfide glutathione (GSSG) were measured following the recycling method described by Anderson (1) with some modifications incorporated from the work described by Rahman et al. (46). Glutathione peroxidase 1 (GPx) activity was measured by spectrophotometry using the method described by Flohe and Gunzler (13). Catalase (CAT) activity was determined by spectrometry using the method described by Claiborne (6). Total superoxide dismutase activity (SOD) was analyzed by spectrophotometry using the method described by Crapo et al. (7).

#### Muscle histology.

A portion of the tibialis anterior muscle was mounted in OCT mounting compound and rapidly frozen in isopentane, and cooled in liquid nitrogen. Eight-micron-thick transverse sections were obtained and stained with hematoxylin and eosin as described previously (31). Fiber sizes were calculated from hematoxylin and eosin-stained histological sections of the FDB muscle using Axiovision 4.0 software to calculate cross-sectional area.

#### Statistical analysis.

Statistical analysis was undertaken using IBM SPSS Statistics version 20. Values are presented as means ± SE; *n* represents the number of fibers in each experiment. Multiple mean comparisons between three experimental conditions at a single time point were analyzed by one-way ANOVA followed by a post hoc least significant difference test. A Student's unpaired *t*-test was used for comparisons between two experimental conditions. Statistical significance was set at *P* < 0.05.

## RESULTS

**Fig. 1.**
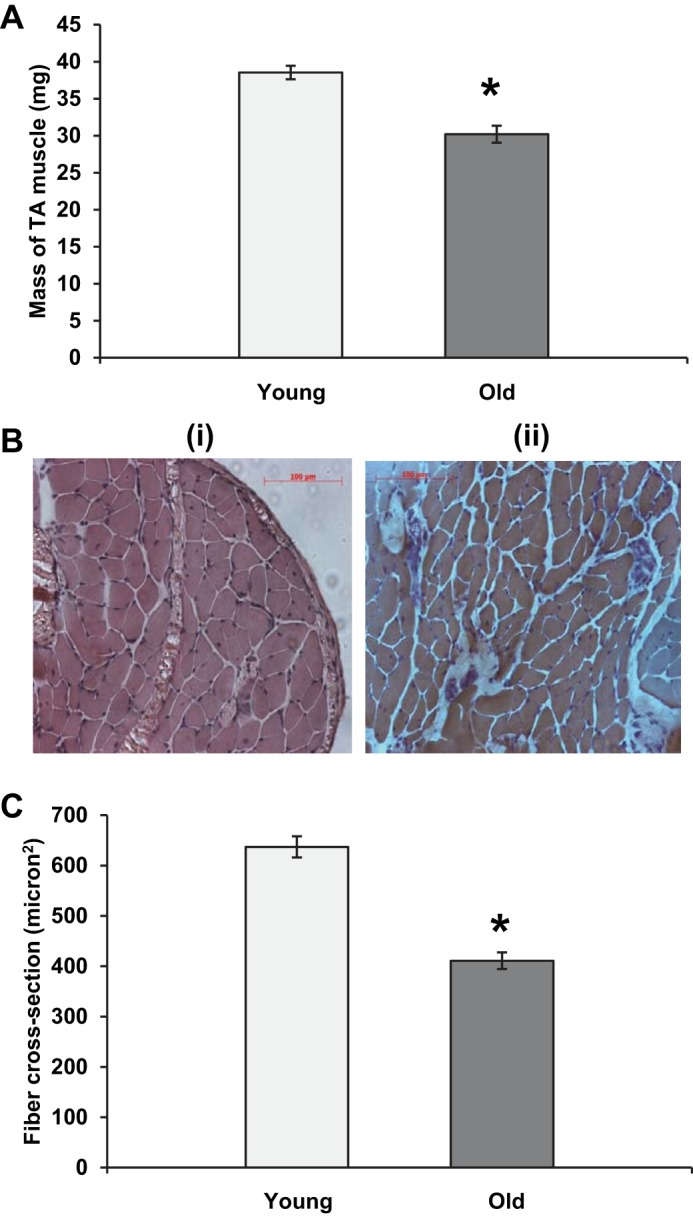
*A:* mass of the tibialis anterior muscle in young and old mice. Data are presented as means ± SE. *P < 0.05 compared with values from young animals, *n* = 50–59. *B:* example of transverse sections from muscles of young (i) and old (ii) mice stained with hematoxylin and eosin. *C:* cross-sectional area of single fibers obtained from the flexor digitorum brevis (FDB) muscle of young and old mice. Data are presented as means ± SE. *P < 0.05 compared with values from young animals, *n* = 100 fibers.

Muscles from the old mice showed the anticipated loss of muscle mass with the tibialis anterior muscle showing ∼15% lower mass in the older mice (Fig. 1*A*). This was associated with only minor structural changes to the muscle. The hematoxylin and eosin–stained cross-sections shown in Fig. 1*B* show an apparent increase in the interstitial space between fibers in muscle from old mice, but no other marked changes in fiber structure were apparent. Measurements of the diameter of fibers obtained from cross-sections of the FDB demonstrated a decrease in fiber cross-sectional area of ∼20% in fibers from old compared with young mice (Fig. 1*C*).

**Fig. 2.**
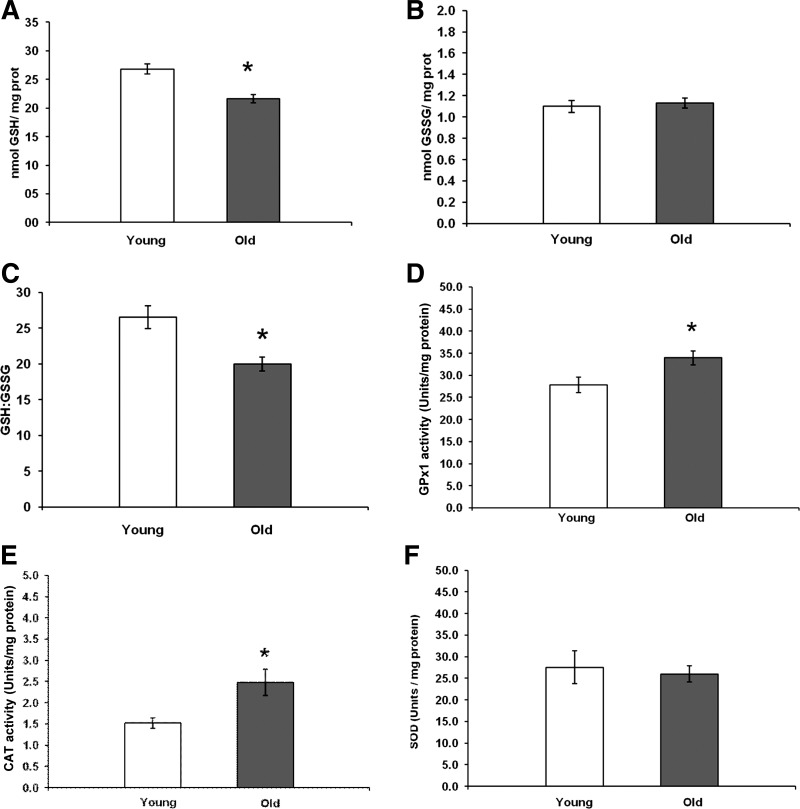
*A:* total glutathione (GSH) content of gastrocnemius muscles from young and old mice. Data are presented as means ± SE. *P < 0.05 compared with values from young animals, *n* = 31–32. *B:* oxidized glutathione (GSSG) content of gastrocnemius muscles from young and old mice. Data are presented as means ± SE, *n* = 31–32. *C:* ratio of total/oxidized glutathione in gastrocnemius muscles from young and old mice. Data are presented as means ± SE. *P < 0.05 compared with values from young animals, *n* = 31–32. *D:* glutathione peroxidase 1 (GPx1) activities of gastrocnemius muscles from young and old mice. Data are presented as means± SE. *P < 0.05 compared with values from young animals, *n* = 8. *E:* catalase (CAT) activities of gastrocnemius muscles from young and old mice. Data are presented as means ± SE. *P < 0.05 compared with values from young animals, *n* = 8. *F:* total superoxide dismutase (SOD) activities of gastrocnemius muscles from young and old mice. Data are presented as means ± SE, *n* = 8.

To place the subsequent measurements of CM-DCFH oxidation in single fibers in the context of age-related changes in regulatory pathways for reactive oxygen and nitrogen species, the total and oxidized GSH content of the gastrocnemius muscles were analyzed together with total GPx, CAT, and SOD activities of the muscle. Data in Fig. 2*A* show that the muscles from aged mice had a significant decrease in total GSH content but no change in the content of oxidized GSH (Fig. 2*B*), and a significantly reduced total glutathione:oxidized ratio (Fig. 2*C*). Muscles from the old mice also showed a significant increase in GPx activity (Fig. 2*D*) and CAT activity (Fig. 2*E*), but no change in total SOD activity (Fig. 2*F*) compared with muscles from the young group. Thus the muscles of this cohort of aged mice showed the changes in regulatory proteins for ROS previously reported (5, 55).

**Fig. 3.**
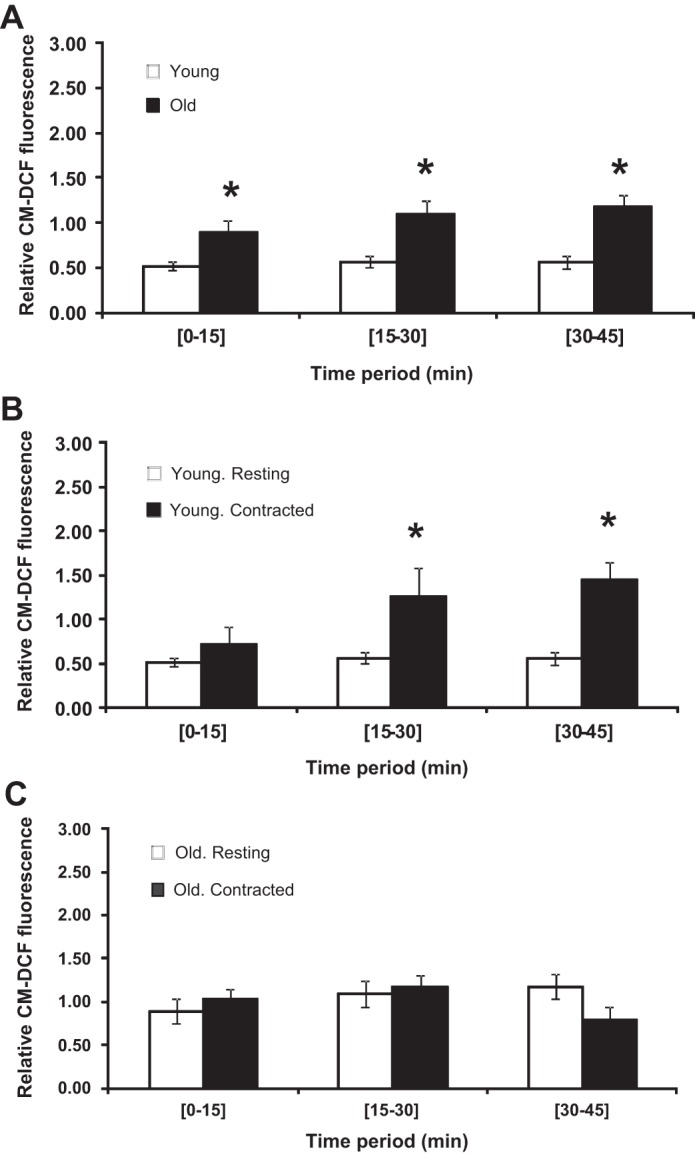
*A:* rate of change in 5-(and-6)-chloromethyl-2′,7′-dichlorodihydrofluorescein (CM-DCF) fluorescence in single quiescent FDB fibers from young and old mice. Data are presented as means ± SE. *P < 0.05 compared with values from fibers of young animals over the same time period, *n* = 15–16. *B:* rate of change in CM-DCF fluorescence in single FDB fibers from young mice that were either at rest throughout the experiment or subjected to a 15-min period of electrically stimulated contractions during the 15- to 30-min time period. Data are presented as means ± SE. *P < 0.05 compared with values from nonstimulated fibers over the same time period, *n* = 15–6. *C:* rate of change in CM-DCF fluorescence in single FDB fibers from old mice that were either at rest throughout the experiment or subjected to a 15-min period of electrically stimulated contractions during the 15- to 30-min time period. Data are presented as means ± SE. *P < 0.05 compared with values from nonstimulated fibers over the same time period, *n* = 16–13.

Figure 3*A* shows the CM-DCF fluorescence from fibers obtained from old and young mice. Fibers remained at rest throughout the 45-min experimental period. CM-DCF fluorescence from the fibers showed the same slow increase over time as previously reported (41), and CM-DCF fluorescence data are presented as relative values (i.e., normalized to the initial fluorescence value measured for each fiber) and expressed as the rate of change over each 15-min measurement period. This mode of expression was previously found to correct for changes in loading between fibers (41). Fibers from both groups showed a consistent rate of oxidation of CM-DCFH over the time course, but the rate of oxidation observed in fibers from old mice was significantly greater than from fibers from young mice throughout the time course. The effect of 15 min of electrically stimulated contractions (commencing at 15 min into the experiment) on fibers from young mice is shown in Fig. 3*B*. This shows that the contractions induced an increase in CM-DCFH oxidation that persisted following the end of the active contractions. In contrast, fibers from the FDB muscles of old mice showed no increase in CM-DCFH oxidation following contractions, although fibers from both groups were observed to contract throughout the 15-min contraction period.

**Fig. 4.**
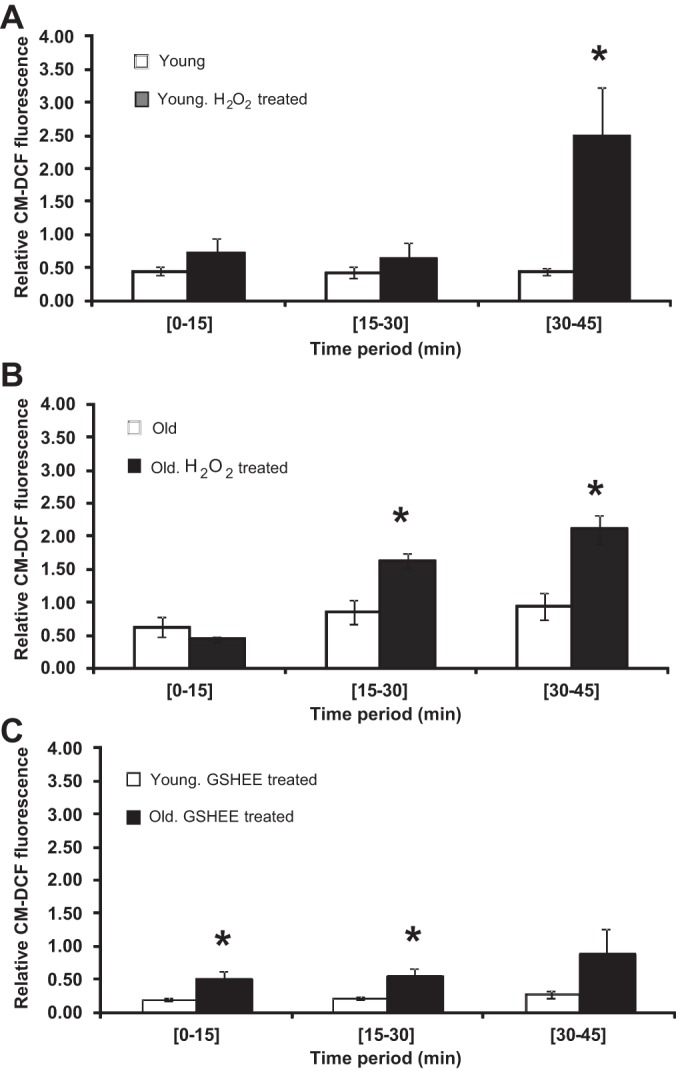
*A:* rate of change in CM-DCF fluorescence in single FDB fibers from young mice that were exposed to 5 μM hydrogen peroxide (H_2_O_2_) during the 15- to 45-min time period. Data are presented as means ± SE. *P < 0.05 compared with values from fibers that were not exposed to H_2_O_2_ over the same time period, *n* = 5–6. *B:* rate of change in CM-DCF fluorescence in single FDB fibers from old mice that were exposed to 5 μM H_2_O_2_ during the 15- to 45-min time period. Data are presented as means± SE. *P < 0.05 compared with values from fibers that were not exposed to H_2_O_2_ over the same time period, *n* = 4. *C:* rate of change in CM-DCF fluorescence in single FDB fibers from young and old mice that were pretreated with 1 mM glutathione ethyl ester (GSHEE) for 18 h prior to loading with CM-DCFH. Data are presented as means ± SE. *P < 0.05 compared with values from fibers from young mice at the same time interval, *n* = 3–4.

To investigate factors that might help explain the differences observed between fibers from young and old mice, groups of fibers were treated with 5 μM H_2_O_2_ during the 15- to 45-min period or pretreated with 1 mM GSHEE for 18 h prior to commencement of the experiment and CM-DCF fluorescence measured. Data in Fig. 4*A* show that H_2_O_2_ induced a significant increase in CM-DCF fluorescence from fibers from young mice only after 30 min of exposure, whereas the CM-DCF fluorescence was increased by 15 min after commencing H_2_O_2_ exposure in fibers from old mice.

When fibers from young and old mice were treated with GSHEE (Fig. 4*C*), both showed a decrease in CM-DCF fluorescence compared with untreated fibers from the same group (e.g., compared with data in Fig. 3*A*), but the significant difference between fibers from old mice compared with fibers from young mice remained, with fibers from old mice continuing to show a higher rate of CM-DCFH oxidation.

**Fig. 5.**
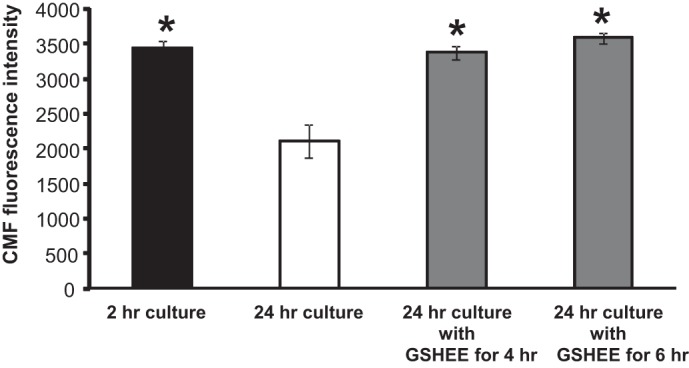
Fluorescence intensities from fibers loaded with 5-chloromethylfluorescein diacetate (CMFDA) as an indicator of intracellular GSH content. Before CMFDA loading, fibers were maintained in culture conditions for *1*) 2 h (*n* = 7); *2*) 24 h (*n* = 5); *3*) 24 h and pretreated with GSHEE (5 mM for 4 h) (*n* = 8); and *4*) 24 h and pretreated with GSHEE (5 mM for 6 h) (*n* = 11). Data are presented as means ± SE. *P < 0.05 compared with values from untreated fibers maintained in culture for 24 h.

To understand the effect of GSHEE treatment on muscle fiber GSH, an additional fluorescent technique was used to assess the relative GSH content of fibers in culture. The CMFDA fluorescence intensity from fibers obtained from young mice was compared following 2 h in culture, 24 h in culture, and 24 h in culture in which fibers were treated with GSHEE for 4 or 6 h. Culture for 24 h was found to result in a decrease in cellular GSH compared with fibers cultured for 2 h (Fig. 5), but this decline was prevented by both treatments of the fibers with GSHEE (Fig. 5).

## DISCUSSION

The main findings of this study were that skeletal muscle fibers from muscles of mice that showed age-related changes in muscle mass also demonstrated increased oxidation of the nonspecific probe for reactive oxygen and nitrogen species, CM-DCFH. Furthermore, a contraction-induced increase in oxidation of CM-DCFH was observed in fibers from young mice, but it was absent in those from the older animals. These data are in accord with the hypothesis that aging is associated with an increased generation of ROS and also provide a potential explanation for the failure of redox-mediated signaling of adaptive responses to contractions that is observed in old animals and humans (21, 55).

Few studies have previously attempted to directly examine the activities of reactive oxygen and nitrogen species in skeletal muscle of young and old rodents, although other researchers have reported increased DCFH oxidation in muscle homogenates from old compared with young mice (2, 3), and increased generation of H_2_O_2_ by mitochondria in permeabilized muscle fibers in situ (42). Our approach has been to utilize the isolated single fiber preparation for these studies because potential contributions for nonmuscle cells are minimized and the data are obtained from intact cells. Homogenization of tissues is recognized to induce release of catalytically active iron from cells that can cause artifactual increases in the apparent ROS activity measured (17). A potential drawback of the use of isolated fibers is that the isolation technique may provide only a subset of the fibers present in the whole muscle and thus, in a study such as this, might lead to isolation of fibers that are not directly comparable from the two groups. While we believe this is theoretically possible, the technique used here leads to the isolation of greater than 80% of the total fibers from the FDB and thus the samples are likely to be representative of the original muscle sampled.

CM-DCFH was used as the ROS-sensitive probe in these studies. This is a widely used approach to provide a general assessment of ROS and some reactive nitrogen species in cells but is also widely acknowledged to be nonspecific and subject to artifact due to its high sensitivity to photooxidation and autoxidation. We have previously described the technical approach used here to minimize photooxidation and autoxidation in monitoring isolated FDB fibers (41), but the data presented here do not allow assessment of which species are active in oxidizing the DCFH. Murrant and Reid (36) previously reported that DCFH in skeletal muscle could be oxidized by H_2_O_2_, hydroxyl radical, NO, and peroxynitrite.

The data we obtained support the possibility that aging leads to an increased generation of reactive oxygen or nitrogen species in skeletal muscle fibers at rest. Skeletal muscle from old mice contains increased levels of markers of oxidative damage (e.g., see Refs. 5 and 55), but the data reported here also show that this tissue has increased activities of catalase and glutathione peroxidase enzymes in old mice. We have previously speculated that these increased activities reflect an attempt by the tissue to adapt to regulate increased H_2_O_2_ (5) and the current data are compatible with this possibility. It has also been previously reported that muscle tissue from old mice contains a decreased total GSH content with an increased proportion of the GSH in the oxidized form (5, 55). This pattern does not occur in all of the major cellular regulatory thiols because the thioredoxin 1 and 2 contents of muscle from aged mice are increased compared with younger animals (10).

To examine whether the increased CM-DCFH oxidation observed in fibers from old mice was potentially due to a diminished ability to detoxify the major ROS, H_2_O_2_, through the reduced muscle GSH content, fibers from both young and old mice were pretreated with GSHEE to elevate muscle GSH content. We have previously demonstrated that this intervention increased fiber GSH content and suppressed the increase in DCFH oxidation that occurs following contractile activity (41). GSHEE treatment of fibers reduced the basal rate of CM-DCFH oxidation by fibers of both young and old mice (Fig. 4*C* compared with Fig. 3*A*), but the difference between fibers of old and young mice remained. Our interpretation of these data is that enhancing the potential capacity of the fibers to scavenge H_2_O_2_ and others had no effect on the difference observed between fibers from young and old mice and hence the change is likely to be due an increased oxidant generation within the fibers from old mice rather than a deficit in cellular GSH content. This conclusion is fully in accord with previous studies that have demonstrated increased release of superoxide and H_2_O_2_ from mitochondria isolated from muscles of old compared with young mice (23, 55).

Studies of the effect of GSHEE on fiber GSH using the CMFDA fluorescent indicator of cell GSH demonstrated that the 24 h of incubation used to allow fibers to firmly attach to the surface of the culture dish induced a drop in fiber GSH content that might potentially influence the apparent fiber CM-DCFH oxidation. This cell culture–induced change in GSH was reversed by treatment with GSHEE.

The data obtained following treatment of fibers from young and old mice with H_2_O_2_ are less clear cut (Fig. 4, *C* and *D*). The increased activities of GPx1 and CAT found in muscles from old compared with young mice (Fig. 2) would be anticipated to increase the capacity of the muscle from old mice to scavenge H_2_O_2_, but the reverse situation was observed. Muscle fibers from old mice showing increased CM-DCFH oxidation within 15 min of treatment, whereas the fibers from young mice did not show any significant changes in CM-DCFH oxidation for a further 15 min. These data suggest that the functional ability of muscle fibers from old mice to remove or scavenge H_2_O_2_ may be compromised compared with that found in fibers from young mice. The relative contribution of different proteins scavenging exogenous H_2_O_2_ is unclear, but this also includes the activities of proteins such as peroxiredoxins in addition to GPx and CAT (17), but unfortunately, it was not possible to examine these proteins in the current study.

In contrast to fibers from young mice, those from old mice showed no increase in DCFH oxidation following contractile activity (Fig. 3). Muscles from adult mice show acute increases in stress proteins and regulatory proteins for ROS following contractile activity, and these adaptive responses have previously been shown to be attenuated in muscle from old mice (5, 54, 56). It has been speculated that this reflects a defect in cellular responses to the ROS generated during contractions (21, 55, 57). The data presented here suggest that the attenuated responses of muscle from old mice to contractile activity may be due to a failure to increase ROS and/or reactive nitrogen species generation in muscle fibers of old mice in response to contractions. A variety of potential sources have been proposed for the ROS generated during contractions (19). Most publications have assumed that the ROS generated in muscle during contractile activity derive from mitochondria [see Jackson (20)], but our recent data have implicated muscle NADPH oxidase(s) in this process (48). Studies of NADPH oxidase in muscle during aging do not appear to have been undertaken. Changes in nitric oxide synthases (NOS) have been reported during aging with a decreased expression of neuronal NOS and an increase in inducible NOS expression both described (16, 49). The net effect of these changes on NO availability is unclear, although Tidball and colleagues (49) have reported that overexpression of NO has beneficial effects on muscle from aging mice.

It has been reported that a proportion of the fibers present in muscles from old mice are functionally denervated (9, 24, 47, 58). It is unclear whether such fibers might have contributed to the data obtained here, although Muller et al. (35) reported that mitochondria isolated from experimentally denervated muscles showed a large transient rise in H_2_O_2_ release. Only fibers that were excitable and contracted on direct electrical stimulation were analyzed in the current study and thus were unlikely to have been denervated for a sustained period. Nevertheless, a role for fibers that had undergone recent denervation in vivo in the pattern of changes observed in the current data cannot be excluded.

### Perspectives and Significance

The data presented indicate that reactive oxygen or nitrogen species are increased in skeletal muscle fibers from old mice at rest, and that these changes are likely due to an increase in endogenous oxidant generation rather than a lack of ROS-scavenging capacity. Surprisingly, the study found no further increase in reactive oxygen and nitrogen species generation in muscle fibers from old mice during contractile activity, a finding that is in stark contrast to the situation in fibers from young mice. These data suggest that the defect in short-term adaptations to contractions that have been extensively reported to occur in old mice may be related to a diminished or absent increase in the muscle generation of reactive oxygen or nitrogen species that accompanies contractile activity in young mice.

## GRANTS

Support for this study was provided by Wellcome Trust Grant 073263/Z/03 and by National Institute on Aging Grant AG020591-06.

## DISCLOSURES

No conflicts of interest, financial or otherwise, are declared by the author(s).

## AUTHOR CONTRIBUTIONS

Author contributions: J.P., D.P., and M.J.J. conception and design of research; J.P., A.V., and D.P. performed experiments; J.P., A.V., D.P., A.M., and M.J.J. analyzed data; J.P., A.M., and M.J.J. interpreted results of experiments; J.P. prepared figures; J.P. and M.J.J. drafted manuscript; J.P., A.V., A.M., and M.J.J. approved final version of manuscript; A.V., A.M., and M.J.J. edited and revised manuscript.
